# Supplementary material for: Impact of Two Phosphorus Fertilizer Formulations on Wheat Physiology, Rhizosphere, and Rhizoplane Microbiota
Source: Int J Mol Sci. 2023 Jun 8;24(12):9879. doi: 10.3390/ijms24129879 (PMC10297904; doi:10.3390/ijms24129879)
Supplement: Supplementary file 1 [file ijms-24-09879-s001.zip › ijms-2385241-supplementary.pdf]

Supplementary material :

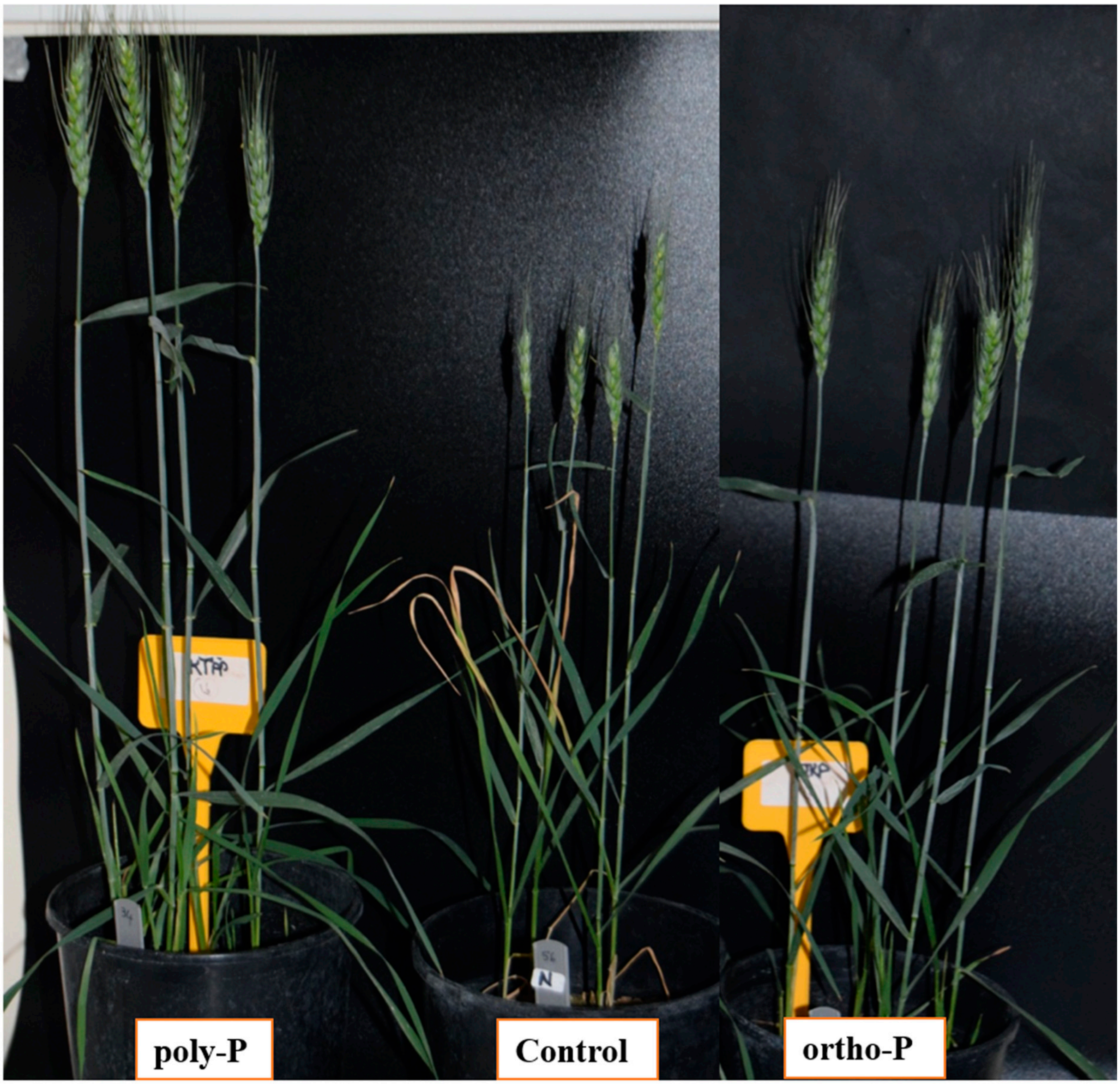

Figure S1 : A photo showing the visual effect of fertilizer treatments on wheat at Z69. Visually, the lack of P in control was reflected in reduced plant length and symptoms of leaf wilting and yellowish spots on the youngest leaves.

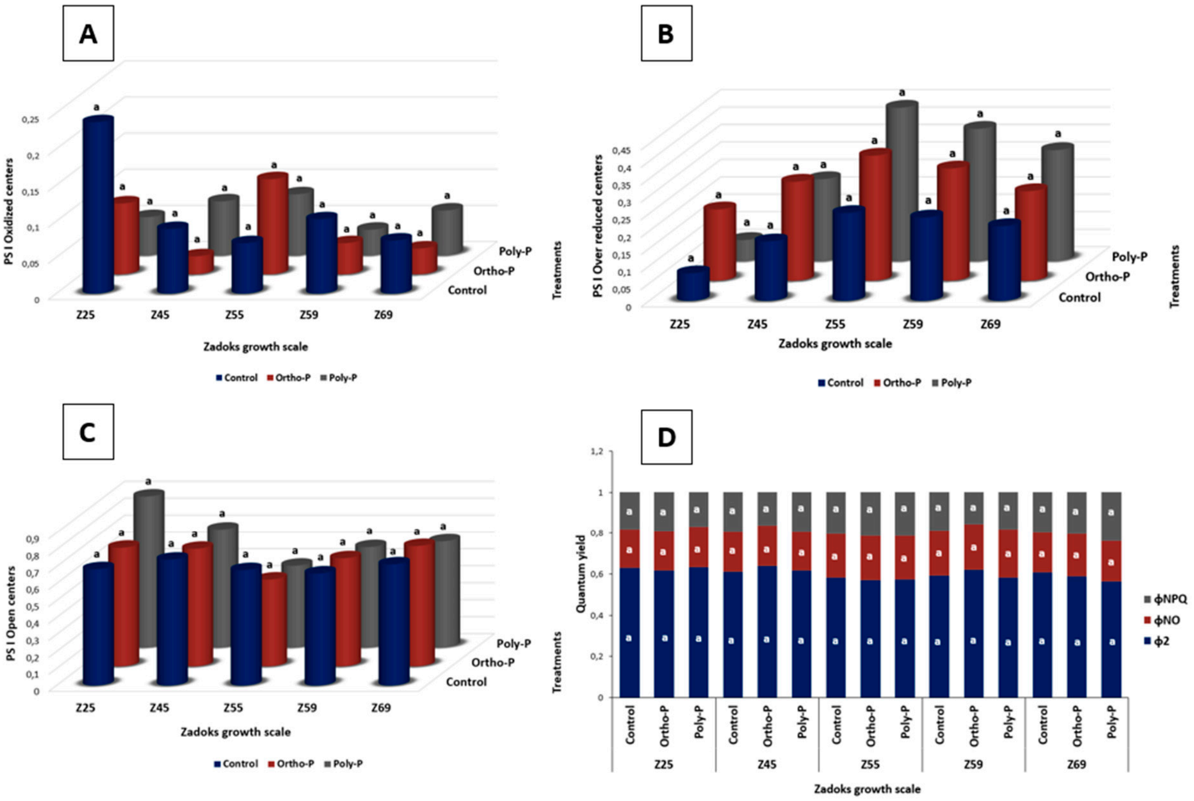

Figure S2: Effect of different Phosphate fertilizers on PSI (A–C) and PSII (D) of bread wheat. Data are expressed as the mean ± SD (*n* = 7). No Significant differences between different treatments are indicated. The statistical analysis was determined by a Tukey's Studentized Range (HSD) test: α = 0.05, *n* = 7 using SPSS

Table S1: Summary table of bacterial ASV

**Table summary**	
Number of ASV	10,916
**Frequency per sample**	
Minimum frequency	529.0
1st quartile	4,023.75
Median frequency	6,535.0
3rd quartile	10,077.25
Maximum frequency	27,451.0
Mean frequency	7,695.482758620689
**Frequency per ASV**	
Minimum frequency	1.0
1st quartile	5.0
Median frequency	17.0
3rd quartile	39.0
Maximum frequency	1827.0
Mean frequency	40.88842066691095

Table S2: Summary table of fungal ASV

Table summary	
Number of ASV	1,251
**Frequency per sample**	
Minimum frequency	103.0
1st quartile	997.75
Median frequency	1,551.0
3rd quartile	2,678.0
Maximum frequency	5,019.0
Mean frequency	1,874.9
**Frequency per ASV**	
Minimum frequency	2.0
1st quartile	12.0
Median frequency	28.0
3rd quartile	72.0
Maximum frequency	5,289.0
Mean frequency	89.92326139088729

Table S3: ANCOM statistical results of ASV for bacterial ASV under ortho-P fertilization at Z69 by group

Strain	*W*
Uncultured Burkholderiales bacterium	212
Uncultured bacterium clone Pyro1	1
Niabella sp. strain Ra-18	1
Uncultured bacterium clone Uday0-27	1
Uncultured Geothermobacter sp.	1
Uncultured Acidobacteria bacterium clone AKYH1411	1
Uncultured bacterium clone B16-TAFD-06	1
Uncultured bacterium clone 12I-L42	1
Uncultured bacterium clone Con65	1
Uncultured Sphingomonadaceae clone3C_19	1
Uncultured Nannocystineae bacterium clone CA29	1
Uncultured bacterium clone CC01f46d04	1
Uncultured bacterium clone Upland_75_7110	1

Table S4: Percentile abundances of ASV for bacterial ASV under ortho-P fertilization at Z69 by group

Percentile	0.0	25.0	50.0	75.0	100.0	0.0	25.0	50.0	75.0	100.0

Group	rhizo plane	rhizo plane	rhizo plane	rhizo plane	rhizo plane	rhizos phere	rhizos phere	rhizos phere	rhizos phere	rhizos phere
Uncultured Burkholderiales bacterium	1.0	1.0	1.0	4.0	13.0	17.0	22.0	23.0	39.0	39.0
Uncultured bacterium clone Pyro1	1.0	1.0	1.0	9.75	36.0	1.0	1.0	1.0	1.0	4.0
Niabella sp. strain Ra-18	1.0	1.0	1.0	4.75	16.0	1.0	1.0	1.0	1.0	2.0
Uncultured bacterium clone Uday0-27	1.0	1.0	1.0	7.50	27.0	1.0	1.0	1.0	1.0	3.0
Uncultured Geothermobacter sp.	1.0	1.0	1.0	8.25	30.0	1.0	1.0	1.0	1.0	6.0
Uncultured Acidobacteria bacterium clone AKYH1411	1.0	1.0	1.0	4.75	16.0	1.0	1.0	1.0	1.0	5.0
Uncultured bacterium clone B16-TAFD-06	1.0	1.0	1.0	8.5	31.0	1.0	1.0	1.0	1.0	1.0
Uncultured bacterium clone 12I-L42	1.0	1.0	1.0	2.0	5.0	1.0	1.0	1.0	1.0	2.0
Uncultured bacterium clone Con65	1.0	1.0	1.0	5.50	19.0	1.0	1.0	1.0	1.0	1.0
Uncultured Sphingomonadaceae clone3C_19	1.0	1.0	1.0	12.0	45.0	1.0	1.0	1.0	1.0	1.0
Uncultured Nannocystineae	1.0	1.0	1.0	5.50	19.0	1.0	1.0	1.0	1.0	1.0

bacterium clone CA29										
Uncultured bacterium clone CC01f46d04	1.0	1.0	1.0	5.25	18.0	1.0	1.0	1.0	1.0	1.0
Uncultured bacterium clone Upland_75_7110	1.0	1.0	1.0	7.0	25.0	1.0	1.0	1.0	1.0	4.0

Table S5: Statistical analysis of P fertilization on microbial diversity performed using Kruskal-Wallis and PERMANOVA

		**Kruskal-Wallis**		**PERMANOVA**	
		Shannon index		Bray-Curtis	
**Factor**	Group	H′	q	Pseudo-F	q
**Bacterial 16S rhizosphere at Z39 (rarefied at 5139)**					
Poly-P	Ortho-P	4,083	0,129	1,129	0,042
	Control	0,083	0,772	0,99	0,526
Ortho-P	Control	0,75	0,579	1,098	0,042
**Bacterial 16S rhizosphere at Z69 (rarefied at 3471)**					
Poly-P	Ortho-P	0,24	0,806	1,107	0,06
	Control	0,534	0,806	1,037	0,192
Ortho-P	Control	0,06	0,806	1,15	0,06
**Bacterial 16S rhizoplane at Z39 (rarefied at 3412)**					
Poly-P	Ortho-P	2,083	0,372	1,119	0,078
	Control	1,333	0,372	1,204	0,078
Ortho-P	Control	0,333	0,567	1,083	0,093
**Bacterial 16S rhizoplane at Z69 (rarefied at 892)**					
Poly-P	Ortho-P	0,24	0,806	0,936	0,875
	Control	0,534	0,806	0,999	0,78
Ortho-P	Control	0,06	0,806	1,034	0,78
**Fungal ITS rhizosphere at Z39 (rarefied at 840)**					
Poly-P	Ortho-P	0,083	0,772	1,461	0,1
	Control	0,75	0,579	0,888	0,73
Ortho-P	Control	0,75	0,579	1,19	0,17
**Fungal ITS rhizosphere at Z69 (rarefied at 915)**					
Poly-P	Ortho-P	0,125	0,72	1,587	0,045

	Control	1,333	0,43	0,953	0,659
Ortho-P	Control	1,12	0,43	1,951	0,045
**Fungal ITS rhizoplane at Z39 (rarefied at 1525)**					
Poly-P	Ortho-P	0,333	0,563	1,161	0,384
	Control	1,333	0,372	1,021	0,384
Ortho-P	Control	2,08	0,372	1,927	0,177
**Fungal ITS rhizoplane at Z69 (rarefied at 1135)**					
Poly-P	Ortho-P	0,75	0,579	0,77	0,62
	Control	0,24	0,624	1,063	0,463
Ortho-P	Control	6	0,042	1,454	0,024

Table S6: ANCOM statistical results of ASVs for bacterial ASV at the Z39 under poly-P fertilization by group

Strain	*W*
Uncultured *alpha proteobacterium clone M1-045*	284
Uncultured bacterium *clone scm94*	1
Uncultured *Burkholderiales bacterium clone4B_11*	1
Uncultured bacterium *clone Con57*	1
Uncultured *Micromonosporineae bacterium clone O:RM-B3*	1
Uncultured *planctomycete clone B05L-1*	1
Taibaiella sp. strain T16R-182	1
Uncultured bacterium *clone ACB10-Oct14*	1
Uncultured bacterium *clone HL05-151*	1
Pedobacter sp. strain F4-32	1
Uncultured Bacteroidetes bacterium	1
Uncultured candidate division TM7 bacterium	1
Uncultured bacterium clone FFCH5499	1

Table S7: Percentile abundances of ASVs for bacterial ASV at the Z39 under poly-P by group

Percentile	0.0	25.0	50.0	75.0	100.0	0.0	25.0	50.0	75.0	100.0
Group	rhizo plane	rhizo plane	rhizo plane	rhizo plane	rhizo plane	rhizos phere	rhizos phere	rhizos phere	rhizos phere	rhizos phere
Uncultured alpha proteobacterium clone M1-045	33.0	55.0	64.5	77.0	110.0	1.0	1.0	1.0	1.0	6.0
Uncultured bacterium	1.0	1.0	1.0	1.0	1.0	1.0	1.0	1.0	1.0	3.0

clone scm94										
Uncultured Burkholderiales bacterium clone4B_11	25.0	26.5	32.0	46.25	74.0	1.0	16.0	22.0	27.0	33.0
Uncultured bacterium clone Con57	1.0	1.0	1.0	1.0	1.0	1.0	1.0	1.0	1.0	26.0
Uncultured Micromonosporineae bacterium clone O:RM-B3	1.0	1.0	1.0	1.0	1.0	1.0	1.0	1.0	1.0	20.0
Uncultured planctomycete clone B05L-1	1.0	1.0	1.0	1.0	1.0	1.0	1.0	1.0	1.0	30.0
Taibaiella sp. strain T16R-182	1.0	1.0	1.0	1.0	1.0	1.0	1.0	1.0	1.0	29.0
Uncultured bacterium clone ACB10-Oct14	1.0	1.0	1.0	1.0	1.0	1.0	1.0	1.0	1.0	34.0
Uncultured bacterium clone HL05-151	1.0	1.0	1.0	1.0	3.0	1.0	1.0	1.0	1.0	30.0
Pedobacter sp. strain F4-32	1.0	1.0	1.0	1.0	1.0	1.0	1.0	1.0	1.0	32.0
Uncultured Bacteroidetes bacterium	1.0	1.0	1.0	1.0	3.0	1.0	1.0	1.0	1.0	13.0
Uncultured candidate division TM7 bacterium	1.0	1.0	1.0	1.0	3.0	1.0	1.0	1.0	1.0	21.0
Uncultured bacterium	1.0	1.0	1.0	1.0	1.0	1.0	1.0	1.0	1.0	4.0

clone FFCH5499										

Table S8: ANCOM statistical results of ASVs for bacterial ASV under ortho-P fertilization at Z39 by group

Strain	*W*
Uncultured beta proteobacterium	430
Uncultured Sphingobacteria bacterium	1
Uncultured Acidobacteria bacterium clone GASP-MA1S1_H11	1
Uncultured bacterium clone SK1_Go0Yyyy6	1
Uncultured Gemmata sp. clone 1c_94826	1
Uncultured delta proteobacterium clone GASP-KC1S2_B06	1
Uncultured bacterium clone lp180	1
Uncultured bacterium isolate Mineral.top.2.1.6_95683	1
Uncultured planctomycete clone F155cmContig21	1
Uncultured bacterium clone C60.10_1318527	1
Uncultured Planctomycetaceae bacterium clone4C_35	1
Uncultured bacterium isolate Mineral.bttm.1.4.1.2_400752	1
Uncultured alpha proteobacterium clone GASP-WC2S1_G08	1

Table S9: Percentile abundances of ASVs for bacterial ASV under ortho-P fertilization at Z39 by group

Percentile	0.0	25.0	50.0	75.0	100.0	0.0	25.0	50.0	75.0	100.0
Group	rhizo plane	rhizo plane	rhizo plane	rhizo plane	rhizo plane	rhizos phere	rhizos phere	rhizos phere	rhizos phere	rhizos phere
Uncultured beta proteobacterium	21.0	29.0	44.0	48.0	75.0	1.0	1.0	1.0	1.0	1.0
Uncultured Sphingobacteria bacterium	1.0	1.0	1.0	1.0	1.0	1.0	1.0	1.0	1.0	4.0
Uncultured Acidobacteria bacterium clone GASP-MA1S1_H11	1.0	1.0	1.0	1.0	1.0	1.0	1.0	1.0	1.0	2.0
Uncultured bacterium clone SK1_Go0Yyyy6	1.0	1.0	1.0	1.0	1.0	1.0	1.0	1.0	1.0	3.0

Uncultured Gemmata sp. clone 1c_94826	1.0	1.0	1.0	1.0	1.0	1.0	1.0	1.0	1.0	6.0
Uncultured delta proteobacterium clone GASP-KC1S2_B06	1.0	1.0	1.0	1.0	1.0	1.0	1.0	1.0	1.0	5.0
Uncultured bacterium clone lp180	1.0	1.0	1.0	1.0	4.0	1.0	1.0	1.0	1.0	1.0
Uncultured bacterium isolate Mineral.top.2.1.6_95683	1.0	1.0	1.0	1.0	1.0	1.0	1.0	1.0	1.0	2.0
Uncultured planctomycete clone F155cmContig21	1.0	1.0	1.0	1.0	4.0	1.0	1.0	1.0	1.0	1.0
Uncultured bacterium clone C60.10_1318527	1.0	1.0	1.0	1.0	5.0	1.0	1.0	1.0	1.0	1.0
Uncultured Planctomycetaceae bacterium clone4C_35	1.0	1.0	1.0	1.0	3.0	1.0	1.0	1.0	1.0	1.0
Uncultured bacterium isolate Mineral.bttm.1.4.1.2_400752	1.0	1.0	1.0	1.0	4.0	1.0	1.0	1.0	1.0	1.0
Uncultured alpha proteobacterium clone GASP-WC2S1_G08	1.0	1.0	1.0	1.0	1.0	1.0	1.0	1.0	1.0	4.0

Table S10: Hypothesis Pairwise PERMANOVA Kruskal-Wallis based on Shannon index testing rhizo-compartment hypothesis.

Variable tested	Dataset	H'	q-value
Rhizo-compartment	Bacteria_poly-P_Z39	4.08	0.04

Rhizo-compartment	Bacteria_Control_Z69	3.84	0.05

Table S11: All hypothesis tests for variations in community structure use permuted multivariate analysis of variance (PERMANOVA) tables based on Bray-Curtis

Variable tested	Dataset	pseudo-F	q-value
Rhizo-compartment	Bacteria_poly-P_Z39	1.24	0.01
Rhizo-compartment	Bacteria_Control_Z39	1.08	0.03
Growth stage	Fungi_rhizoplane_Control	1.30	0.04
Growth stage	Bacteria_rhizosphere_poly-P	1.24	0.02
Growth stage	Bacteria_rhizosphere_ortho-P	1.25	0.02

Table S12: ANCOM statistical results for bacterial ASV in rhizosphere under poly-P fertilization

Strain	*W*
Pseudoxanthomonas sp. strain RL94	295
Uncultured bacterium clone 2005-MA-5-100207	294
Uncultured *alpha proteobacterium* clone M1-045	293
Bacterium strain GSD10062	261
Luteimonas sp. CB 286455	249
Lysobacter sp.	246
Uncultured bacterium clone RS-Apr-08	144
Uncultured *Lysobacter* sp. Clone Plot17-C11	21
Uncultured *Luteimonas* sp. clone S5_I30	14
Uncultured bacterium clone 6-22	5
Uncultured soil bacterium clone S1P4029	5
Uncultured *alpha proteobacterium* clone 2181	5
Rhizosphere soil bacterium isolate RSI-24	5

Table S13: Percentile abundances of ASVs for bacterial ASV in rhizosphere under poly-P fertilization by group

Percentile	0.0	25.0	50.0	75.0	100.0	0.0	25.0	50.0	75.0	100.0
Group	Z39	Z39	Z39	Z39	Z39	Z69	Z69	Z69	Z69	Z69
Pseudoxanthomonas sp. strain RL94	1.0	1.0	1.0	1.0	16.0	14.0	15.0	17.0	30.0	56.0
uncultured bacterium clone 2005-MA-5-100207	1.0	1.0	1.0	1.0	18.0	8.0	11.0	16.0	16.0	23.0
Uncultured *alpha proteobacterium* clone M1-045	1.0	1.0	1.0	1.0	12.0	9.0	10.0	10.0	10.0	11.0

Bacterium strain GSD10062	1.0	1.0	1.0	1.0	24.0	7.0	8.0	11.0	17.0	18.0
Luteimonas sp. CB 286455	1.0	1.0	1.0	1.0	32.0	11.0	15.0	16.0	18.0	27.0
Lysobacter sp.	1.0	1.0	1.0	1.0	52.0	12.0	18.0	21.0	21.0	27.0
Uncultured bacterium clone RS-Apr-08	1.0	1.0	1.0	1.0	14.0	6.0	12.0	18.0	19.0	33.0
Uncultured *Lysobacter* sp. Clone Plot17-C11	1.0	1.0	1.0	1.0	1.0	9.0	10.0	19.0	20.0	38.0
Uncultured *Luteimonas* sp. clone S5_I30	1.0	1.0	1.0	1.0	1.0	6.0	16.0	19.0	20.0	32.0
Uncultured bacterium clone 6-22	1.0	1.0	1.0	1.0	17.0	1.0	1.0	1.0	1.0	1.0
Uncultured soil bacterium clone S1P4029	1.0	1.0	1.0	1.0	14.0	1.0	1.0	1.0	1.0	1.0
Uncultured alpha proteobacterium clone 2181	1.0	1.0	1.0	1.0	15.0	1.0	1.0	1.0	1.0	1.0
Rhizosphere soil bacterium isolate RSI-24	1.0	1.0	1.0	1.0	42.0	1.0	1.0	1.0	1.0	1.0

Table S14: Pairwise pseudo-F test based on Bray Curtis distance matrix revealed that wheat growth stage significantly contributed to bacterial community differences in the rhizoplane.

Data set	Group 1	Group 2	pseudo-F	q-value
Bacteria_rhizoplane_poly-P	Z39	Z69	0.97	0.63
Bacteria_rhizoplane_ortho-P	Z39	Z69	1.14	0.02
Bacteria_rhizoplane_control	Z39	Z69	1.10	0.11
